# Posterior Mediastinal and Cutaneous Back Hemangiomas in Infants: A New Association

**DOI:** 10.1055/s-0040-1721408

**Published:** 2021-05-12

**Authors:** Amr AbdelHamid AbouZeid, Shaimaa Abdelsattar Mohammad, Heba Gomaa Aly, Iman Ahmed Ragab

**Affiliations:** 1 Department of Pediatric Surgery, Faculty of Medicine, Ain Shams University, Cairo, Egypt; 2 Department of Radiodiagnosis, Faculty of Medicine, Ain Shams University, Cairo, Egypt; 3 Department of Pediatrics, Faculty of Medicine, Ain Shams University, Cairo, Egypt

**Keywords:** hemangioma, magnetic resonance imaging, paravertebral, vascular tumors

## Abstract

Infantile hemangiomas (IHs) are common vascular tumors. In most cases, a benign course with favorable outcome can be anticipated. IH typically present as cutaneous lesions either with a localized or diffuse segmental distribution. Segmental hemangiomas in the face may be associated with brain and cardiac anomalies (PHACES syndrome), whereas airway involvement has been reported to be associated with hemangiomas in the “beard” area. Multiple cutaneous hemangiomas may be associated with visceral hemangiomas (commonly in the liver).

In this report, we present a new association where deep paravertebral hemangiomatous lesions were observed to be associated with cutaneous back hemangiomas in two consecutive cases.

## Introduction

Infantile hemangiomas (IHs) are common benign vascular tumors. 1 IHs are characterized by a natural history where most tumors would go into spontaneous involution by the age of 7 years. 1 2 Generally, an excellent prognosis can be anticipated with few reported complications. 3 Exceptionally, very large tumors may cause hemodynamic instability in infancy. Lesions in specific locations are more prone to cause complications: periorbital hemangiomas may obstruct the field of vision, and subglottic lesions can obstruct the airway. 4 5 Expectant treatment is the rule for the management of uncomplicated IHs. When indicated, systemic corticosteroids and more recently propranolol have been successfully used to induce/accelerate tumor regression. 2

IHs typically present as cutaneous lesions either with a localized or diffuse segmental distribution. 2 Segmental hemangiomas in the face may be associated with brain and cardiac anomalies (PHACES syndrome), whereas airway involvement has been reported to be associated with hemangiomas in the “beard” area. 2 5 Multiple cutaneous hemangiomas may be associated with visceral hemangiomas (commonly in the liver). 2

In this report, we present a new association where deep paravertebral hemangiomatous lesions were observed to associate cutaneous back hemangiomas in two cases.

## Case 1

**Fig. 1:**
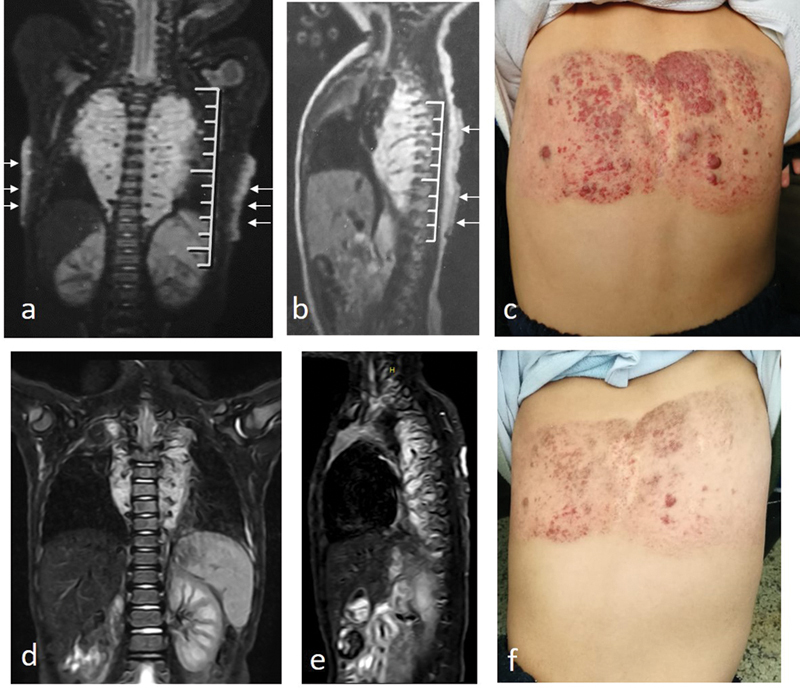
First case of a boy with cutaneous back and posterior mediastinal hemangiomas. ( **a,b** ) magnetic resonance imaging ([MRI] coronal and sagittal) performed at the age of 3 months (before treatment) demonstrating the presence of large intrathoracic posterior mediastinal (paravertebral) hemangioma (bright T2 signal with characteristic intralesional flow voids). *White arrows* are pointing to cutaneous back hemangioma in ( **a** ) and ( **b** ). ( **c** ) The appearance of back hemangioma at the time of referral (2 years old). ( **d–f** ) Follow-up at the age of 3 years demonstrating marked regression of the mediastinal lesions on MRI ( **d,e** ), as well as regression of the cutaneous back hemangioma after propranolol treatment.

**Fig. 2:**
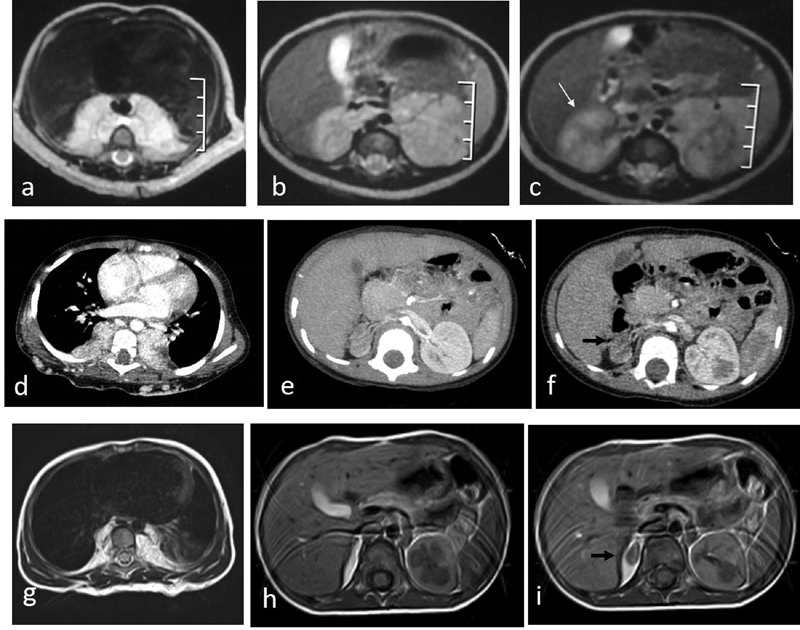
First case of a boy with cutaneous back and posterior mediastinal hemangiomas. ( **a–c** ) Magnetic resonance imaging (MRI) study performed at the age of 3 months before treatment. The mediastinal mass was seen in ( **a** ) as bilateral symmetrical mass, pre- and paravertebral in location, compressing the adjacent posterior aspects of both lung lobes and encasing adjacent parts of the aorta. The retroperitoneal component was seen in ( **b** ) and ( **c** ) in the suprarenal region partially encasing the upper pole of both kidneys. ( **d–f** ) Computed tomography (CT) study performed at presentation to our clinic (age of 2 years). Note the regression of the paravertebral masses; however, there was atrophy of the right kidney ( *black arrow* in **f** ), whereas it was present in the first study at the age of 3 months ( *white arrow* in **c** ). ( **g–i** ) MRI study performed at follow-up after treatment (at the age of 3 years) demonstrating marked regression of the deep hemangiomatous lesions (posterior mediastinal and retroperitoneal). Note atrophy of the right kidney ( *black arrow* ).

A boy was born with a flat back hemangioma. Rapid increase in the size of the cutaneous lesion (proliferative phase) was noticed by the parents at the age of 1 month. The parents revealed a history of oral propranolol treatment that started at the age of 3 months and discontinued later at the age of one and a half years. At the age of 2 years, the patient was referred to our vascular anomaly clinic with a previously performed magnetic resonance imaging (MRI) study at the age of 3 months ( Fig. 1a–c ). Besides the cutaneous lesion, the MRI study demonstrated the presence of large posterior mediastinal and upper abdominal retroperitoneal soft tissue masses. The mediastinal mass was seen bilateral symmetrical pre- and paravertebral in location, compressing the adjacent posterior aspects of both lung lobes and encasing adjacent parts of the aorta ( Fig. 2a ). The retroperitoneal component was seen in continuity with the mediastinal mass in the suprarenal region partially encasing the upper pole of both kidneys ( Fig. 2c, d ). The deep paravertebral lesions (mediastinal and retroperitoneal) showed typical MRI features of IH: intermediate signal on T1 and bright T2 and STIR signal with characteristic intralesional flow voids ( Fig. 1 a,b ). No evidence of intraspinal or transforaminal extension, hilar or mediastinal lymphadenopathy, or pleural or pericardial sac collection could be detected. Apart from mild microcytic anemia, the hematological parameters of the case were normal. A follow-up CT scan was ordered upon referral (age of 2 years) that showed partial regression of the deep paravertebral lesions ( Fig. 2d–f ); however, atrophy of the right kidney was noted (confirmed by the absence of activity on renal isotopic scan). Upon referral, oral propranolol was represcribed to the patient (2 mg/kg/day in three divided doses) and continued till the age of 3 years. The cutaneous lesion showed progressive regression with propranolol ( Fig. 1c, f ). Similarly, the deep lesions showed marked regression on follow-up imaging ( Fig. 1d, e ; Fig. 2h–i ). Regarding the atrophy of the right kidney, the patient was directed to follow-up at the pediatric nephrology clinic.

## Case 2

**Fig. 3:**
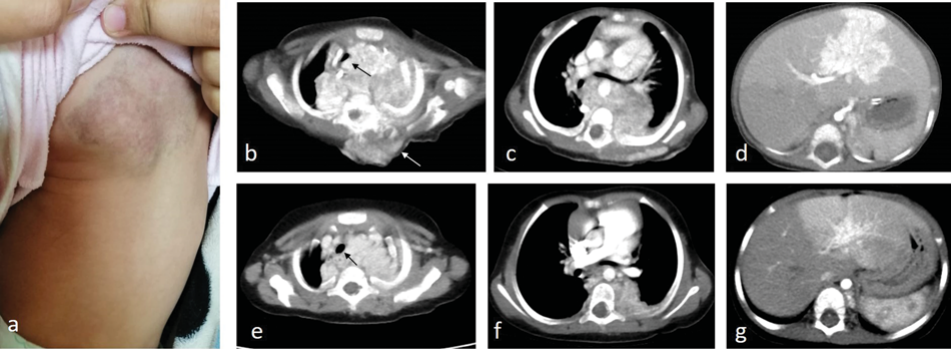
Second case of a girl with cutaneous back and posterior mediastinal hemangiomas. ( **a** ) The appearance of the back hemangioma. ( **b–d** ) CT with intravenous contrast demonstrating the hemangiomatous lesions in the subcutaneous ( *white arrow* ), posterior mediastinal, and liver. The mass is seen in ( **b** ) with intraspinal extension and causing tracheal deviation ( *black arrow* ), while is seen encasing the aortic descending aorta in ( **c** ). A similar lesion with intense contrast enhancement was seen in the left hepatic lobe in ( **d** ). ( **e–g** ) Follow-up CT demonstrating mild regression in the upper mediastinal lesion in ( **e** ) with less compression on the airway, which is still displaced to the right ( *black arrow* ), whereas mild-to-moderate regression of the lesion is seen in ( **f** ) and ( **g** ).

A 6-month-old girl presented with respiratory distress and a diffuse back hemangioma ( Fig. 3a ) that was small at birth. Chest X-ray showed tracheal deviation to the right. To avoid the need for general anesthesia, a CT scan rather than MRI was ordered showing a large posterior mediastinal mass extending from the retropharyngeal space to the level of T11, with intense contrast enhancement in the arterial phase. The mass was seen encasing the aortic arch and descending aorta with intraspinal extension and causing tracheal deviation ( Fig. 3b, c ). No sizable mediastinal or hilar adenopathy could be detected. A similar lesion with intense contrast enhancement was seen in the left hepatic lobe ( Fig. 3c ). Hematological investigations showed no abnormality. Systemic corticosteroids were prescribed for the respiratory distress, and a CT-guided biopsy was taken from the posterior mediastinal mass that confirmed the diagnosis of hemangioma. Oral propranolol was added with gradual tapering of corticosteroids, yet the patient had recurrent attacks of bronchitis and pneumonia with bronchospasm necessitating pediatric ICU admission. Interruption of propranolol and resumption of systemic corticosteroids (prednisolone 2 mg/kg/day) was therefore applied for a period of 6 months. The patient had delayed gross motor milestones; neurological examination showed mild hypotonia with normal deep tendon reflexes mostly attributed to prolonged steroid courses. Corticosteroid treatment was discontinued again, and the patient returned to oral propranolol after stabilization of her chest condition. Almost complete regression of the cutaneous lesion was observed. A follow-up CT showed regression of the hepatic lesion ( Fig. 3g ) and mild-to-moderate regression of the deep paraspinal lesion ( Fig. 3e, f ).

## Discussion

Reviewing the literature, one can still find confusing terminology in reports on mediastinal hemangiomas when authors described venous malformations using the old terminology of cavernous haemangiomas. 6 7 Truong et al reported on the successful use of propranolol in the treatment of a life-threatening subglottic and mediastinal IH in a female infant with no evidence of cutaneous lesions. 8 In this report, we describe in two consecutive patients a new association between diffuse cutaneous back hemangioma and the presence of deep paravertebral hemangiomatous lesions, which, to the best of our knowledge, has not been reported before.

The paravertebral hemangioma caused mediastinal compression in one of our cases who suffered from breathing difficulties at the age of 6 months. Propranolol was discontinued for bronchial obstruction, and systemic corticosteroids were used instead. Maybe a combination of both drugs would have achieved a synergistic effect. 9 More invasive treatment modalities can be used, such as intralesional steroid/bleomycin injection; also, sirolimus may be tried for refractory cases. 10 In the first case, the paravertebral lesions were at a lower level and thus did not cause mediastinal compression. However, we have some evidence that the retroperitoneal component of the lesion might have obstructed the right renal vessels during the proliferative phase of the tumor resulting in atrophy of the right kidney.

One of the two cases was also associated with a hepatic focal lesion displaying characteristic radiological features of hemangioma. Although a similar pathological diagnosis of IH might be suspected, focal hemangiomas of the liver are more commonly congenital (negative for Glut-1). 11

This new association may prove to be important from two aspects. First, a diagnostic confusion may arise from the presence of the posterior mediastinal (paravertebral) mass. 12 Knowing about the presence of such association besides the typical imaging features of hemangioma can unmask this confusion 13 ; otherwise, invasive diagnostic biopsy will be needed. The other aspect is related to the morbidity associated with such paravertebral hemangiomatous lesions. The rapid proliferation of hemangioma in this critical location encasing the aorta and its main branches may result in significant morbidity. Another potential morbidity is related to intraspinal extension and posterior mediastinal compression manifestations (mediastinal syndrome). Taking in consideration the potential comorbidities of IH in this location, we strongly recommend early establishment of maximum treatment upon diagnosis of such cases with closer follow-up.

## Notes

New Insights and the Importance for the Pediatric Surgeon We present a new association where deep paravertebral hemangiomatous lesions were observed to be associated with cutaneous back hemangiomas in two cases. This association may prove to be important from two aspects: differential diagnosis of the posterior mediastinal (paravertebral) hemangioma, and another aspect related to the morbidity associated with such location.
